# Stakeholder perspectives on the ethico-legal dimensions of biobanking in South Africa

**DOI:** 10.1186/s12910-021-00645-z

**Published:** 2021-07-01

**Authors:** Shenuka Singh, Keymanthri Moodley

**Affiliations:** Centre for Medical Ethics and Law, Department of Medicine, Faculty of Health Sciences, Stellenbosch University, Tygerberg, South Africa

**Keywords:** Stakeholders, Biobanking, Ethics, Biobanks, Biorepositories

## Abstract

**Background:**

Biobanking provides exciting opportunities for research on stored biospecimens. However, these opportunities to advance medical science are fraught with challenges including ethical and legal dilemmas. This study was undertaken to establish perspectives of South African stakeholders on the ethico-legal dimensions of biobanking.

**Methods:**

An in-depth exploratory study was conducted with 25 purposively selected biobankers, clinicians, researchers, postgraduate students in biobanking research, and research ethics committee (REC) members in South Africa. Potential study participants were recruited through known hubs for biobanking in the country, online searches and the snowball sampling technique. A semi-structured face-to-face or Skype interview was arranged. Data was analysed using thematic analysis.

**Results:**

The emergent themes included: inconsistency in understanding consent models, disconnect between biobank researchers and biosample donors, inadequate processes to support re-consenting minors, inconsistent governance processes for biobanking research; challenges with sample and data sharing, and suboptimal strategies for benefit sharing and return of results. Biobanking practice in general appeared to be inconsistent and fragmented. While the need for consent in research is explicitly outlined in legislative documents, some respondents were unclear on the type of consent model to apply in biosample collection. They also reported inconsistencies in research participants’ understanding of consent. Furthermore, these respondents’ own understanding of consent and consent models were dependent on where they were positioned in biobanking practice (roles occupied). Respondents were unsure about the process to follow to re-consent child participants once the age of majority (≥ 18 years) was reached. It was not surprising that consent was identified as one of the major ethical challenges in biobanking practice. In certain settings, some respondents reported suboptimal governance processes for sample collection. Participants were generally unsure about how to operationalise benefit sharing and how to approach the idea of returning results to research participants and biobank donors.

**Conclusion:**

The study findings indicated inconsistencies in stakeholder understanding of ethico-legal considerations related to biobanking in South Africa. A need for ongoing ethics capacity development among stakeholders was identified. Improving understanding of the ethics of biobanking could be facilitated by acknowledging the disconnect created by biosamples in the relationship between biobank researchers and donors.

## Background

The conduct of biomedical research involving the collection, use, storage and export of well-annotated human biological material has generated considerable debate in South Africa, given the associated ethical and legal complexities [1–4]. While the National Health Act (Act 61 of 2003) (NHA) makes provision for the regulation of tissue banking and transplantation in South Africa [5], the provision for biobanking in the Act, is notably absent [6]. The lack of clear legislation in this field creates a loophole for different interpretations of the law and given South Africa’s history of exploitation and human rights violations, greater scrutiny and more robust and explicit guidance and legislation are required on the ethical and legal aspects of biobanking. Despite South Africa’s transition to democracy (post 1994), social and economic inequalities persist creating a fertile ground for health inequities that could open a gateway for continuing exploitation. One such case is the recent publicized dispute between an academic institution in South Africa and the Wellcome Sanger Institute in the UK over the alleged commercialization of transferred biological material and data, intended and consented for research purposes only [7–9]. To elaborate, a South African research site initially shared samples with a university in the United States (US) through a research collaborative initiative. This was done with the necessary export permits in place for the cross border transfer of samples and with the affected research participants’ consent. The shared samples were subsequently transferred to the Wellcome Sanger Institute for research-related genome analysis, as part of the agreed material transfer agreement (MTA) between the South African institution and the university in the US. The contention arose when the samples were allegedly further shared by the Wellcome Sanger Institute with a third party for the creation of gene chips (also known as ‘microarrays’ for rapid genetic testing) but without the original South African university’s knowledge nor with consent from research participants. Additionally, the South African university claimed that there was no data sharing agreement with this third party and demanded the return of the shared samples from the Wellcome Sanger Institute [7, 10, 11].

The extent to which researchers balance scientific imperatives in knowledge advancement through biobanking research while upholding ethical obligations to research particpants, affected communities and participating institutions, remains unclear [3, 12]. This is of particular interest given that communities such as the San people of sub-Saharan Africa have shown greater awareness of exploitation in research and have responded with a community-driven ethical code of conduct for research [13].

Biobanking practice in South Africa ranges from small scale research projects located within academic and research-based institutions to large scale archived diagnostic samples stored within teaching hospitals; established biobanks in public/academic settings; private non-profit registeries and profit-based cord and stem cell tissue banks [6]. The multi-disciplinary nature of biobanking research adds to this debate given that stakeholders (both direct and indirect) emerge from diverse disciplines such as science, medicine, law, genetics, genomics, bioethics, technology and bioinformatics [14]. This diversity reflects a complex landscape in biobanking-related activities but little is known about how stakeholders engage with context-specific ethical challenges in biobanking research in South Africa. Some of the known ethical challenges include participants’ rights to privacy and confidentiality, autonomy, return of results, benefit sharing and secondary use of biospecimens and data [3, 15]. The extent to which researchers and funders engage in commercialization of biosamples in public and academic biobanking efforts remains largely unknown.

Given this incomplete picture of stakeholder involvement in the context of legislative lacunae, the purpose of this study was to understand stakeholder perspectives on ethico-legal considerations in biobanking practice in South Africa. This study forms part of a larger project on the ethical, legal and social issues associated with genomic and biobanking research [16–19].

## Methods

This was an in-depth exploratory study using a qualitative approach to engage with stakeholders involved in biobanking in an attempt to seek their perspectives on ethico-legal considerations in contextualised settings. In this study, stakeholders were defined as individuals (biobankers, researchers, clinicians, nurses) involved in the operationalization and/or oversight of managing stored biological material, or in the ethics review of such related research (research ethics committee/REC members). The study excluded stakeholders such as policy makers, funders and research participants.

There are specific groups of individuals involved in biobanking practice and health research ethics related to biobanking in the country, therefore a purposive sampling technique was used to identify the various hubs for biobanking practice and research ethics review in the country. All identified individuals in the biobanking hub were also requested to link the researchers with other stakeholders involved in biobanking and research ethics review. Thus, a snowball sampling technique was also used. Additionally, online searches and literature reviews were conducted to further identify stakeholders beyond the existing network of people involved in biobanking to ensure a wide representation of the study sample.

The study population thus comprised biobankers, clinicians, researchers, postgraduate students in biobanking research, and REC members. A semi-structured face-to-face or Skype interview was conducted with the identified stakeholders (n = 25) (Table 1).

**Table 1:** List of respondents

Interviewee	Position/Experience
1	Researcher, biobanker, REC member University A
2	Researcher, University B
3	Clinical care, nurse, University B
4	REC member, research ethics teaching University B
5	Clinician, researcher, biobanker, University B
6	Researcher, biobanker, University B
7	Retired: Research ethics
8	Clinician, researcher, biobanker, University B
9	Researcher, research ethics, University B
10	Researcher, REC member, University A
11	Retired: research ethics, REC member
12	Clinician, researcher, biobanker, University B
13	Researcher, clinician, REC member, University A
14	Researcher, REC member, University A
15	REC member, researcher, University A
16	Researcher, REC member, University A
17	Researcher, postgraduate student, University A
18	Researcher, biobanker, University A
19	Researcher, post graduate student, University A
20	Researcher, postgraduate student, University A
21	Researcher, research ethics, University D
22	REC member, University D
23	Research ethics, University D
24	REC member, University D
25	Researcher, biobanker, University C

It was noted that study participants could occupy more than one role, such as biobanker, researcher, REC member and clinician as reflected in Table 1. Letters of invitation for participation in the study were sent to all identified individuals in these hubs. All interested individuals were requested to contact the researchers if they expressed interest in participation in the study. The interview was arranged, based on the participant’s availability and willingness to participate in the study, and after written informed consent was obtained, during the period: January 2019 to August 2019. The interview was approximately 30–60 min in duration and was digitally recorded.

The interview focused on the following topics: ethical challenges facing biobanking practice in South Africa and stakeholder perspectives on improving understanding of research in this area. Further probing during the interview focused on participants’ understanding of consent, return of results and benefit sharing. These probes were included because the identified issues are poorly defined in existing South African guidelines. Given the general inability of some study participants to engage with the identified issues during the interview, a follow-up interview (via email) was conducted with 4 study participants with expertise in biobanking, to seek clarity on benefit sharing and return of results for biobanking research.

Thematic analysis was used to analyze the textual and contextualised opinions on biobanking practice to produce arguments that could reinforce or undermine the study participant’s position or point of view [20]. Narratives from the interviews were coded and analyzed using ATLAS.ti 8 Windows software package. Data was first recorded verbatim and a transcript was created for each interview. This was cleaned before the analytical process. Familiarization of the data entailed reading through a descriptive narrative of each participant’s perspective. Initial coding of data was conducted to build a coding frame. Codes were then defined, reviewed, collated and organised into code groups. Each code group was further examined for patterns and emergent themes. These themes were named and further examined to identify patterns and linkages between the themes. The researchers also identified quotes in the study participants’ transcripts that were congruent with the overarching themes. The emergent themes from data analysis were further reviewed and then compared with the available literature. This contributed to transferability of the data collected and allowed for extrapolation of the research findings to other similar settings. A co-coder was used and themes were identified independently and then compared and agreed upon. Member verification was also conducted to ensure that the collected data was a true reflection of the participant’s statements on biobanking practice.

## Results

Given the heterogeneity of biobanking practices, it was not surprising that there was a wide range of experiences and perceptions among study participants. The themes arising from this study included inconsistency in understanding consent models, disconnect between biobank researchers and biosample donors, inadequate support for the re-consenting process for minors involved in providing samples for biobanking; inconsistency in governance processes for biobanking research; challenges with sample and data sharing, and suboptimal strategies for benefit sharing and return of results.

Biobanking practice in general appeared to be inconsistent, fragmented and poorly defined. Perceptions on the state of biobanking varied from well organized facilities (according to biobankers) to ad hoc unclear processes for collection and distribution of biospecimens (according to researchers, post graduate students, REC members and clinicians).

In response to the question: *What is the state of biobanking practice in the country?*, more than two thirds of the study sample (researchers, clinicians, biobankers and REC members) believed that the climate of biobanking in South Africa was punctuated by a general lack of understanding among researchers, clinicians, research participants and communities on the exact nature and purpose of biobanks, biobanking and biorepositing of samples.

We are talking about secondary use,… data and sample transfer across borders and these are complex things, especially in our setting in Africa, taking into account all our different cultures and traditions. And also the lack of understanding, you don’t really want to say lack of education because even the educated person still has the opinion that biobanking is just [about] storage in freezers, which it is not (6).

The notion of respect for research participants and communities emerged as a key ethical consideration in biobanking practice.

### Inconsistency in understanding consent models

In response to the question: *What are the ethical challenges in biobanking practice in the country?* some of the reported ethical challenges included the consent process for obtaining the biosample (specifically in relation to research participants’ understanding of the research purpose or their anxiety associated with the extraction of biosamples) and re-consenting processes for samples taken from children (< 18 years of age). The study findings suggested that some respondents had challenges with the process of obtaining consent from research participants but failed to identify the different consent models in biobanking research. Given that study participants occupied multiple roles in  biobanking practice (researcher, clinician, biobanker, REC member), these multi-fold challenges further highlighted the complexity in biobanking research.

From a legal perspective, the National Health Act (NHA) (Act 61 of 2003) replaced the Human Tissue Act No 65 of 1983 in South Africa and now regulates the use, collection and disposal of human biological samples such as tissues, blood and blood products, and gametes [5]. Research participants’ rights to autonomy are enshrined in the NHA:  Sect. 71(1) [5]. Similarly, Chapter II (Sect. 14) of the South African Constitution highlights the right to privacy as a fundamental right. More recently, South Africa adopted the Protection of Personal Information Act 4 of 2013 (POPIA), which came fully into effect from July 2020. Section 13(1) of POPIA (2013) outlines that personal information [which would include biometric information and DNA analysis] must be collected for a ‘specific, explicitly defined and lawful purpose’. Section 15 of the Act specifies that further processing of samples for research must be compatible with the original purpose for which it was collected [21]. Thus POPIA provides a constitutional right to privacy and highlights the need for data security, participant confidentiality and access to information [21].

While POPIA specifies the need for specific consent, broad consent and other consent models are supported in national guidelines such as the South African Department of Health’s Ethics in Health Research: Principles, Processes and Structures (DOH 2015) [22]. These two documents could be seen as contradictory thus could contribute to a lack of clarity in terms of the type of consent to be used in biobanking. Given that POPIA has only been fully implemented in 2020 and that legislative processes still allow for an additional year for public comment and stakeholder input, there is no doubt that there is a need for alignment of national processes and guidelines related to consent for research purposes.

While the legal position for consent in research is clearly defined (as articulated by NHA), the type of consent model to be used in biobanking remains vague. It was therefore not surprising that there were varied responses in this study to the type of consent required for storage of biospecimens but more importantly there was inconsistency among study participants in understanding the type of consent model to be used in the collection of biosamples.

The divergent understandings of consent further articulated a contrast between the legal position and what actually happens in biobanking practice. Study participants (researchers, clinicians, REC members) indicated that the storage of biosamples for unknown future use had ethical implications. The notion of consent, however was seen more as a technical process without due consideration to the different models of consent and implications for the biobank research participants’ autonomy and privacy. This is of particular importance given the dual roles that a clinician/researcher could occupy and that risks associated with future unknown use of biosamples could differ from those in clinical settings.

In response to the questions, *What are some of the consent models used in biobanking? What could be the associated ethical challenges?*, broad consent, dynamic and tiered consent models were identified, but at least six out of the twenty-five (6/25) participants were unaware of the different consent models in biobanking research.

The big ethical challenge that we have is that people are very used to broad consent and haven’t really been held to account for what participant information they provide, and now with the POPI Act coming up that’s not going to be acceptable anymore. I think people are pushing… researchers don’t like to make a change when they are used to something being the way it is, and I hear lots of moaning about how this is going to shut down genomics (21).

### Disconnect between biobank researchers and biosample donors

This theme indicated that understanding the notion of consent or the models of consent used, was very much dependent on where the respondent was positioned in biobanking practice, and how this could possibly influence ethics oversight in biobanking. A respondent at the forefront of the primary collection site was able to articulate the process of obtaining consent from the research participant. A respondent dealing with the stored biosample had little interaction with the actual research participant or donor.

When people work with the sample they don’t look at its purpose, they just see it as colourless liquid and I think that it would be nice [to remember] that this actually belongs to a human being, and we are just the custodians of the sample, and that we should have respect when we use it (12).

Respondents (researchers, clinicians, REC members) identified a disconnect between the collected biospecimen and the individual who provided the biosample, where the focus was on the biosample without due consideration that the sample was obtained from a human being. Part of the reported challenges included concerns about researchers who dealt only with the biospecimens and were not directly involved in the clinical collection point at the interface with research participants or biosample donors. Respondents (researchers, postgraduate students, a nurse, clinicians, biobankers, REC members) thought that respect had to apply to both the individual research participant and his/her collected biospecimens.

Thus the physical distance between research participants or biosample donors (at the sample collection point) and the biobank could paradoxically create a distance in the relationship between biobank researchers and donors. This suggests that ethics oversight could be diluted because of the lack of direct accountability. This distance could be increased when the samples are shared with other researchers, thus suggesting a further dilution of ethics oversight and accountability.

### Consent for biobanking specimens from minors

Some respondents (researchers, clinicians, REC members) also highlighted ethical challenges that could arise when specimens taken from minors (< 18 years of age) are collected for biobanking purposes. The NHA stipulates that parental or legal guardian consent is required for child participants (< 18 years of age) in research. Ethical dilemmas can arise when children reach the age of independent consent (≥ 18 years). Should the child participant (now an adult) be re-consented?

The NHA also made provisions for the creation of the National Health Research Ethics Council (NHREC) which is a statutory body responsible for research ethics oversight as well as the registration of RECs in South Africa. According to the NHA, Sect. 72(1); responsibility is given to RECs (that are registered with the NHREC) within South Africa to review and approve such research, when all criteria for participant protection are met [5].

Additionally, the South African Department of Health’s Ethics in Health Research: Principles, Processes and Structures (DoH 2015) [22] highlights that ministerial permission is required for the ‘removal of biological materials from mentally ill persons; or materials taken from a minor that are not naturally replaceable and that no foetal biological material except for umbilical cord progenitor cells may be collected from anyone’ [22]. While the guideline mentions re-consent in general, there is no clarity on how this should be implemented. While these stipulated conditions for the removal of biological material from mentally incapacitated persons or minors are meant to protect the identified populations, the use of such nomenclature could also create possible confusion. One such example could be that while blood is replaceable, such procedures are also invasive.

The study findings suggested a further disconnect between the legal position and study participants’ awareness of these support structures. Attention should also be drawn to the inadequacies of national guidelines such as Ethics in Health Research: Principles, Processes and Structures (DOH 2015) [22] to provide clarity on the process of re-consenting minors for biobanking research.

Study participants (researchers, clinicians, REC members) raised questions on whether and how re-consenting should occur once these participants reached the age of independent consent (age 18 and above in South Africa). Study participants were unsure on how to re-consent these participants nor was there reported clarity on the process of re-consenting.

“…in our kind of context especially in studies that involve minors who may reach the age of majority during the sample storage period, how actually is that managed because it is difficult to often contact participants down the line? How do researchers ensure that they are able to get consent from minors when they reach the age of majority? (24).

RECs should be an important resource for researchers requiring clarity on ethical issues related to biobanking, provided that these committees have the capacity and skills mix to handle such queries.

### Inconsistencies in governance processes for biobanking research

There were inconsistencies reported in the governance of biobanking in the country. Biobankers believed that biobanking initiatives were good and reported efforts made to strengthen biobanking governance in South Africa through collaborative networks such as ISBER and initiatives driven by the H3Africa consortium. There was a perception that biobanking governance in these settings was underpinned by best practices.

Concurrently some biorepositing efforts appeared unstructured and poorly defined (those reported by postgraduate students), thereby suggesting inadequate governance processes in certain settings. This clearly implied differences in the nature of biobanking efforts across different settings and that the governance (collection and processing) of biospecimens was very much dependent on the contextualized setting. Study participants (postgraduate students) reported challenges encountered when travelling from academic institutions to community-based health facilities for the extraction, and transport of collected biosamples. There were vague processes in place to support collection of biospecimens under these conditions. Some of the reported challenges encountered  by postgraduate students included incomplete clinical records for documenting information, incorrect processes for labelling of biospecimens, and unclear tracking systems for biospecimens. Transportation of biospecimens was identified as a challenge specifically when postgraduate students were involved in the collection and transfer of biosamples for research purposes. Study participants (postgraduate students) reported having no option but to use public transport facilities to ferry biosamples from the collection points (hospitals) to the clinical laboratories (university settings) for analysis. Apart from the hazards of transporting fluids such as blood, the integrity of such biosamples could become questionable when transported in sub-standard conditions. This casts serious doubt on the value of collecting biosamples under such conditions.

Some students without transport do have a problem. It is unethical I think, for HIV samples to be carried in glass bottles using public transport… because the hospitals are far away from where they are based [university]. They are not allowed to analyze any of the blood in the hospitals. The laboratory is always busy so we have to carry the samples back to campus laboratories (19).

The implications were that some stakeholders (in this case postgraduate students) were not treated fairly as researchers in biobanking. This raises ethical and legal concerns.

### Challenges with sample and data sharing

From a legal perspective, the South African Material Transfer Agreement of Human Biological Materials (SA MTA 2018) is a ‘a contract governing the transfer of materials between organisations and /or institutions’ and provides a framework for ‘providers and recipients of the biological material for use in research or clinical trials under the auspices of the Health Research Ethics Committees’ in South Africa [23]. Additionally South African legislation clearly prohibits commercialization of body parts or blood and blood products [5].

While the SA MTA (2018) [23] provides a legal solution to the sharing of biospecimens with third parties, some respondents (REC members, researchers) highlighted the limitations of the current document.

It is un-operational at this moment in time. Now having a MTA is correct. We are absolutely in favour of that, we think it is absolutely appropriate but the way the offering was provided was terrible, we can’t operationalize that (22).

An MTA should clearly specify the roles and responsibilities of each identified stakeholder, including the sender and the recipient. It is equally important that such a document gets ‘buy-in’ or support from all stakeholders, including research participants. One respondent used the Malawian experience to articulate how legislation was developed in that country to respond to the uncontrolled flow of biospecimens out of the country.

There were all sorts of rumours that some of the researchers from the West who had collected samples from Malawi and shipped them to the West, were selling those samples to other researchers to do their studies, in a way Malawi did not benefit from… at the end of the day, it became like a commercial activity and those that were benefitting were actually the researchers that came from the West, to collect those samples from Malawi (9).

One of the potential unanticipated outcomes of sample and data sharing could be possible commercialization of biosamples, as articulated in the above quote. Apart from this comment, none of the other respondents indicated that commercialization of biosamples was an ethical issue to consider in biobanking. It should be noted that the interview schedule did not include any probes on the commercialization of biosamples.

### Suboptimal strategies for benefit sharing

There was inconsistency among respondents on the notion of benefit sharing in biobanking, in response to the following questions: “What is your understanding of benefit sharing? How should this be implemented?”.

You could be [involved in biobanking] for cancer research and if you come up with a cure or something happens from what you have found, there should be some benefit for the community from which the samples came (16).

There were strong views on parachute or helicopter research where respondents reported that researchers from the global north, used developing countries as biosample extraction depots, with very little consideration for benefit sharing or developing local capacity in biobanking research.

We often see people doing benefit sharing [efforts] in communities. So people come in from outside Africa and they say, we will do a workshop for you. They have decided what the contents of the workshop are, they have decided that we need that workshop and often the workshop is repeated stuff that we have [been] taught here long ago, or it is not relevant. So there is lots of benefit sharing as collaborators and participants in South Africa but very little, real benefit sharing that I see (21).

Other views alluded to statements in the consent document that should explicitly outline the biobank’s position on benefit sharing. One respondent further pointed out that an academic biobank whose sole purpose was to facilitate sharing of biosamples for research purposes would not be involved in benefit sharing efforts. The responsibility for devising benefit sharing plans, in that scenario, would lie with the individual researchers who use the stored biosamples. Thus the notion of benefit sharing requires a wider conversation involving all stakeholders.

I think benefit sharing should not be a single ethics committee responsibility. I think that needs a national discussion around biobanking per se and how we as a country look to biobanking principles and benefit sharing (22).

Such conversations should include community and other stakeholder engagements as well.

### Suboptimal processes for return of results

Similarly challenges were noted with some respondents’ understanding of return of results or the practicality of this process. In response to the question, *What is your understanding of return of results?*, less than a third of the study population (researchers, clinicians, biobankers, REC members) were able to articulate some understanding of the notion of return of results.

We don’t do enough to interrogate return of results with research for future samples. I am trying to think if we have asked about incidental findings being returned and I have to admit, we don’t interrogate that enough (24).

For those respondents who could articulate an understanding of return of results, it was clear that researchers had a moral obligation to return results that were clinically actionable. However, there were no clear processes for how this should be done, that is, should results be returned to the biobank to return to the participant or returned directly to the participant? Clearly this issue needs to be interrogated in the governance frameworks for biobanking practice.

It was also seen as necessary to define the target group and devise a strategy for return of results, as suggested by one respondent:

One must define the target groups that could or should benefit from the return of results. The kind of results to be returned must be appropriate for the target group. The following is not a complete list:

(i) The scientific community – publications, congress proceedings, internet.
(ii) General public or society – publication of biobank activities in non-scientific publications, e.g., public websites, annual reports, university-based e.g., Faculty publications, open media, information sheets, brochures.
(iii) Selected target groups, including clinicians, patients, participants, communities, e.g. CANSA, postgraduate students doing biobank science projects, undergraduate students who visit our biobank. (5)

### Improving biobanking practice

In response to the question: “How do we improve biobanking practice in the country?”, respondents (biobankers) highlighted the value of proper governance structures with identified leadership, clear roles and responsibilities for sample collection, best practices for sample storage, access and sharing. Overall the highlighted themes suggested a review of biobanking governance so that some minimum standards can be set across the various settings where the study participants are based.

The need for and the role of ongoing educational efforts were identified as possible ways of improving understanding of biobanking in the country.

I think the knowledge gaps starts for many people at a very basic level. Just understanding what is a biobank and what a biobank is all about, the different types of biobanking. For example, research ethics committees need to know what to look for when they have to approve a biobank application. Patients. and the public need to understand what biobanking is all about. It would be important to identify different needs for knowledge at different levels. So [a] general then focused knowledge distribution and training component is important. Target specific role players and stakeholders (5).

## Discussion

Emergent themes in this study suggested an inconsistent understanding of ethical considerations in biobanking. This finding is supported by Pawlikowski et al., who also reported some inconsistencies in biobanking practice in Poland [24]. The reported inconsistencies in biobanking practice across different settings in South Africa suggested that there are significant gaps in stakeholder (researcher, biobanker, clinician, nurse, postgraduate students, REC member) awareness in knowledge of ethics required for biobanking.

### Dilution of ethics accountability in biobanking

Another theme highlighted the disconnect between biobank researchers and biosample donors and that this divide could create a distance in the relationship between the biobank and research participants or donors. This in turn could impact and possibly dilute ethics accountability and oversight on the part of biobank researchers. Improving understanding of the ethics of biobanking could be facilitated by acknowledging the distance created by biosamples in the relationship between the biobank researchers and donors. This distance is increased when samples are shared globally and sensitivity to ethical oversight may be further diluted. The Sanger case highlighted earlier, is one such example of the consequences of lapses in ethical oversight. In particular, the Sanger case [7, 10, 11]. Illustrates the complexities in biobanking efforts even when data protection measures are in place.

### Heterogeneity in biobanking practice

Previous empirical research on the ELSI of biobanking in South Africa highlighted the need to understand the context of biobanking practice and the need to engage with stakeholders such as researchers and communities [3, 12, 16–19]. This study goes beyond previous empirical evidence in that while there is an acknowledgement of the complexities in biobanking practice, ‘ELSI literature on biobanking has largely focused on issues associated with a biobank’s relationship to its contributors’, [and] little attention has been placed on the *people* involved in the operationalization and management of the biobank [25]. This study reiterated the persistent heterogeneity in biobanking practice reported in a previous study [12] but further illustrated the different challenges that could occur, depending on where the study participants were based and what their roles were.

### Multiplicity in stakeholder roles in biobanking

The multiple roles played by study participants (researcher, biobanker, clinician, REC member) reflected the added layer of complexity to biobanking practice. These roles, e.g. clinician/ researcher could potentially be contradictory, specifically with regards to the ethical requirements from a clinical and biobanking research perspective. An example of such possible contradiction could be where the clinician responsible for clinical care also needs to collect biospecimens for unknown research purposes. How does the clinician /researcher navigate the ethical requirements for different purposes? What supportive processes are in place to guide the clinician/researcher in these dual roles?. How does this differ when the researcher is in a biobank interacting with biospecimens only or when a researcher is only using biospecimens obtained from a biobank in the absence of a relationship with the donor? What ethics capacity development initiatives are in place? This clearly indicates that more effort needs to be invested in recognizing these multiple roles that stakeholders occupy.

We will now focus on some of the specific ethico-legal aspects highlighted in the study findings.

### The need for consistency in governance processes

While biobankers expressed good governance practices, the reported challenges by postgraduate students in collecting and transporting biological biospecimens raise both ethical and legal concerns. Soo et al.also argue that collection and storage of biosamples that are not aligned to best practice models could threaten the value of biobanking research [26]. The reported challenges in this study reiterate the need for national legislation on a minimum set standards for the collection of biosamples [3, 6, 27] irrespective of the context of sample collection. This includes the need for oversight in protecting participants’ interests and rights. Participants’ privacy and confidentiality should be adequately addressed irrespective of whether the sample is collected and processed in a biobank or simply collected from routine clinical procedures and re-purposed for research.

The need for robust governance structures and processes in South Africa is also supported by other empirical studies [12, 19] and given the current lack of ‘regulation or governance of biobanking’ from a legal perspective in South Africa, more effort is required to strengthen legislation on biobanking in the country. This study highlighted that these are persistent challenges that have not been adequately addressed, although POPIA (2013) does provide some direction in this area. Thus, the need to increase stakeholder awareness of the ethical implications of sample and data storage and sharing, and the value of proper governance structures and processes to facilitate access and sharing of biosamples, should be an urgent consideration.

### Consent

A clearly stipulated governance framework or lack thereof could have an impact on the nature and value placed on obtaining consent from research participants. Respondents reported challenges in operationalizing the consent process with research participants (where perceived limited research participant understanding was cited). This coupled with inconsistencies in respondents’ understanding of consent models illustrated the need to re-focus attention on how consent is operationalised in biobanking.

The study findings suggested that understanding of consent becomes more diluted when the stakeholder is further from the research participant, as illustrated earlier. This would have important ethical implications for how biosamples are processed, stored and shared. However, the study findings also demonstrate that a clearly defined consent process does not negate the other ethical issues related to biosample collection and storage. Engagement with the consent process, including the selection of an appropriate consent model would be critical for biobanking research. It is therefore necessary to unpack the challenges highlighted in this study with regards to the use of specific and broad consent.

### Broad consent versus tiered consent

It should be noted that respondents in this study were merely asked about the ethical concerns in biobanking research. No specific model of consent was presented to the respondents, yet those who were aware of consent models, were mostly able to discuss the merits of a broad consent model. This could possibly be attributed to the way in which broad consent facilitates data and sample collection for researchers at the expense of suboptimal participant autonomy as well as the prominence accorded to broad consent in the biobanking literature.

One interviewee in this study highlighted the skewed dependence on the use of broad consent in biobanking research. While this model of broad consent is generally accepted [28] and supported in some circles in biobanking practice in South Africa [29], there is ongoing debate on its relevance and appropriateness. The lack of clear directives for biobanking research in current legislation and guidelines, create opportunities for different interpretations of the legal position in South Africa, as alluded to earlier. Staunton et al*.,* argued that Sect. 15 of POPIA (2013) allows for broad consent to be taken at the outset and that specific consent would ‘stifle current and future research and innovation’ because of the challenges associated with re-contacting and re-consenting participants [29]. Thaldar and Townsend counter argued that POPIA (2013) allows for specific consent only and while ‘genomic research is in the public interest’, the ‘protection of research participants’ privacy rights in their genomic information’ cannot be undermined by broad consent processes [30].

Similarly, questions are raised as to whether broad consent is truly informed and whether it allows for more or less researcher accountability to provide maximum participant information [7, 31, 32]. Stakeholders (such as biobankers, clinicians, researchers, REC members). need to consider the following: does broad consent include possible future commercialization of the shared biosamples? To what extent does the notion of benefit sharing become eroded when broad consent is used? What are researchers’ ethical obligations for benefit sharing when broad consent is used?

Despite these debates, researchers will need to comply with the Protection of Personal Information Act (2014) [21], given that the legal requirements for consent in research will dictate the model of consent to be used in South Africa. This creates an obligation to act in the best interests of research participants/biospecimen donors, to ensure an authentic consent process is followed and that participant privacy and confidentiality are upheld [33]. A balance is therefore required for an appropriate consent process that would ensure protection of individual and community rights while maximizing optimal research benefits [33, 34].

There is a need for ‘ongoing [consent] processes [that] should address how each individual participant may be assured autonomy, agency and individualised choice’ [35]. A combination of consent options, articulated in a tiered consent model might be a plausible compromise as proposed by some authors for genomic research in Africa [36, 37]. A tiered consent model could optimize participant autonomy while promoting scientific progress, despite its known shortcomings (such as resource constraints for tracking individual participant’s choices [38]. A tiered consent model could address some of the persistent challenges that have been identified by this study. More importantly a discussion around consent options in biobanking, both from a legal and ethical dimension could facilitate ongoing stakeholder engagement so as to identify ways to overcome these challenges. This highlights the need for ongoing ethics capacity building that should be included in the governance framework of the biobank.

### Re-consent involving minors

Another theme emerging from this study related to re-consenting minors who reached the independent age of consent (≥ 18 years). Respondents reported not knowing how to deal with re-consent related to children involved in biobanking research. This study finding is consistent with other studies that outline the need to consider the rights of the child participant [39, 40]. While a re-consenting process should be put in place for children who reach the age of independent consent so as to uphold their rights and interests [39, 41], stakeholders should also receive educational support and information on how to operationalize this process.

### Benefit sharing

Although the study findings indicated inconsistencies in understanding and defining the concept of benefit sharing, some respondents were able to identify possible strategies that could be beneficial to individuals and communities. This finding is consistent with an earlier study that also highlighted the need for stakeholders to engage in benefit sharing initiatives [12]. Benefit sharing in research remains contentious [42, 43]. This suggests that the nature and scope of benefit sharing requires more attention [44] and that further research is required in this area. One possible source of guidance for benefit sharing could be Sect. 7 of the SA MTA (2018). Although the reported shortcomings of the document are noted, this section does highlight the need for contracting parties to identify ways of sharing benefits that should be negotiated before the transfer of such material [23]. The San Code on Research Ethics also highlights benefit sharing initiatives such as co-creation of research for knowledge production and shared opportunities for skills transfer and local capacity building [13].

Additionally a tiered consent model could allow for the individual and the community’s ‘specific choices to be voiced on benefit sharing’ [8], however the limitation of this approach should be noted, specifically in cases where communities have little bargaining power on issues related to benefit sharing.

Benefit sharing is particularly important in the context of commercialisation where participants in previous studies expressed a desire to share in profits generated [3]. Again, there is a need for ongoing stakeholder awareness on the scope and nature of benefit sharing to ensure that this does not remain an aspiration but rather is implementable in local settings.

### Commercialization of biospecimens

Although the SA MTA (2018) indicates in Sect. 17.2 that ‘neither party may assign or cede any benefit, obligation or interest it may have in this agreement to any other person without the prior written consent of the other party and the approval of the HREC’ [23], research ethics guidance documents are not explicit about commercialisation. The fact that commercialization of biospecimens (involving researchers) was not highlighted as a prominent ethical concern in this study, is problematic in light of the legislative loopholes with respect to material transfer in South Africa and the recent case of the alleged misuse of South African data and samples by the Wellcome Sanger Institute in the UK [7, 10, 11]. This incident has highlighted the global challenges with the governance of data and sample sharing when this occurs in the absence of respect for research participants, research collaborators and collaborating institutions [8].

The Sanger Institute debacle could have triggered further investigations into commercialisation in biobanking research by the affected research collaborators. The findings of such investigations are yet to be disclosed publicly. In our study, one can only speculate that commercialization was not fully interrogated from an ethics perspective, because South African researchers could have a disproportionate dependence on collaborators in developed countries for funding and resources or this could possibly be due to the asymmetrical power relationships that evolve in some international collaborations [45, 46]. Possible commercialization of biosamples as an ethical concern, requires further debate and research in South Africa. It is imperative that a well-thought out governance framework for research related biobanking consider mechanisms to protect participants from possible exploitation [47, 48] or commercialization of shared biosamples. This needs to be supported by ongoing efforts to strengthen stakeholder awareness so as to mitigate possible commercialization of biospecimens.

### Return of results

Interestingly, return of results could be regarded as a form of benefit sharing too. The reported challenges in the return of results in this study are consistent with other published findings in that there is an urgent need to identify ways to operationalize the concept of return of results to individual participants [49–51]. Returning results to research participants is seen as showing respect for participant autonomy and interests [52]. However, the notion of benefit sharing and return of results must also take into account the challenges that can occur once data has been anonymized and cannot be traced back to the research participants.

Overall the inconsistencies in participants’ understanding of ethical considerations in biobanking, as reported in this study, are not unique. Other studies also highlighted ongoing challenges in stakeholder engagement with ethical issues. Goisauf et al., reported that participants in their study sought information (from national and international guidelines) and support (from helpdesks) to design and improve informed consent documents [53]. Nansumba et al., added that given the ‘disparities in research infrastructure and capacity’ in Lower- and Middle-Income Countries (LMIC) settings, there is a compelling need for education related to the science and ethical dimensions of  biobanking [54]. Similarly, de Vries et al*.,* highlighted the need for REC members to engage with training efforts for the review of genomic and biobanking research [2]. These comments suggest that stakeholder skills and preparedness for working in the world of biobanking requires ongoing review and increased access to educational courses and workshops on skills development.

### The need to build ethics capacity in biobanking

We further contend that to focus on expanding and improving the environment for biobanking without building adequate capacity in the very people entrusted to engage and manage this process, remains an ethical concern. Additionally, there are legal implications when a workforce has gaps in knowledge related to the ethical aspects in the identified field. One of the important legal requirements for health-related research in South Africa is the need for researcher competence that is complemented by ongoing basic training in research ethics [5, 22] that would ensure awareness of  ethics in general as well as ethics compliance in practice. Such training should also highlight the need for further consultation, communication and collaboration with other relevant stakeholders in biobanking. These could include community engagement efforts, legal and ethics related advice and science communication. All of these issues further reiterate the need for a multidisciplinary approach to biobanking.

One finding to emerge from this study is the need for specific training that is tailored for the different layers and levels of stakeholders involved in biobanking. Stakeholders require ongoing support and training to engage meaningfully with the ethical dimensions and legal implications of biobanking. There is an urgent need to review the availability and accessibility of training opportunities in order to close this identified knowledge gap. A review of the current platforms for stakeholder training is required as well as an assessment of the appropriateness of these programmes to meet the diverse needs of individuals. Training programmes in biobanking that are tailored to respond to the ethical and legal challenges in local settings could be of greater value to stakeholders, thus international training courses that are not contextualized to local settings may not be appropriate, and a one ‘size fits all’ training approach should definitely be avoided.

There is thus an urgent need to explore opportunities such as investments in educational training that could form part of capacity development to improve stakeholder understanding of these important, crucial implications for successful and sustainable biobanking initiatives and practices in South Africa.

## Limitations of the study

These findings provided a unique lens into the complex nature of biobanking research that occurs in various formal and informal settings. Given that this study focused on eliciting stakeholder perspectives on ethico-legal ethical considerations in biobanking research in South Africa, its generalizability may be limited to similar LMIC settings. Another limitation is that not all possible stakeholders formed part of the study. More research is required to determine community and research participants’ perspectives, as stakeholders in biobanking research. Future research in this area should also include policy makers, national regulators and civil society members. This study did not sufficiently explore the concept of commercialisation in biobanking. Future research to unpack the concept of commercialisation in biobanking in South Africa is necessary.

## Conclusion

Overall, the study findings indicated inconsistencies in stakeholder understanding of ethico-legal considerations related to biobanking in South Africa. This necessitates the need for ethics capacity building for the identified stakeholders in biobanking. One plausible suggestion could be training opportunities that are tailored to meet the local needs of these stakeholders.

## Data Availability

The datasets used and/or analyzed during the current study are available from the corresponding author on reasonable request. This is subject to participants’ expressed permission to share anonymized interview transcripts with a third party.

## Abbreviations

ELSI: Ethical, Legal and Social Implication
NHA: National Health Act (Act 61 of 2003)
MTA: Material transfer agreement
POPIA: Protection of Personal Information Act 4 of 2013
REC: Research ethics committee
